# Impact of Dietary Supplementation with Goji Berries (*Lycium barbarum*) on Microbiological Quality, Physico-Chemical, and Sensory Characteristics of Rabbit Meat

**DOI:** 10.3390/foods9101480

**Published:** 2020-10-16

**Authors:** Marta Castrica, Laura Menchetti, Claudia M. Balzaretti, Raffaella Branciari, David Ranucci, Elisa Cotozzolo, Daniele Vigo, Giulio Curone, Gabriele Brecchia, Dino Miraglia

**Affiliations:** 1 Department of Health, Animal Science and Food Safety “Carlo Cantoni”, Università degli Studi di Milano, Via Celoria 10, 20133 Milan, Italy; marta.castrica@unimi.it (M.C.); claudia.balzaretti@unimi.it (C.M.B.); 2 Department of Agricultural and Agri-Food Sciences and Technologies, University of Bologna, Viale Fanin 46, 40138 Bologna, Italy; laura.menchetti7@gmail.com; 3 Department of Veterinary Medicine, University of Perugia, Via San Costanzo 4, 06126 Perugia, Italy; raffaella.branciari@unipg.it (R.B.); david.ranucci@unipg.it (D.R.); dino.miraglia@unipg.it (D.M.); 4 Department of Agricultural, Food and Environmental Sciences, University of Perugia, Borgo XX Giugno, 74, 06121 Perugia, Italy; elisa.cotozzolo@libero.it; 5 Department of Veterinary Medicine, University of Milan, Via dell’Università 6, 26900 Lodi, Italy; daniele.vigo@unimi.it (D.V.); giulio.curone@unimi.it (G.C.)

**Keywords:** rabbit meat, goji berries, microbiological quality, sensory characteristics

## Abstract

Forty-two New Zealand White rabbits (*n* = 21/group) were fed with two different diets: a commercial diet (control group) and a diet supplemented with goji berries (3% *w*/*w*). After slaughtering, the effect of dietary supplementation on microbiological, physico-chemical, and sensory characteristics of the rabbit loins, packed in an oxygen-permeable package, was evaluated at 6 h post mortem (day 0), after 4 and 10 days of refrigerated storage. No relevant results were obtained for pH and total volatile basic Nitrogen (TVBN) values but with regards to the color, some significant differences were observed between the groups. The goji berries (GBs) dietary supplementation had positive effects by reducing thiobarbituric acid reactive substances (TBARS) values in all the observations (*p* < 0.001). Moreover, microbiological results showed that the supplementation had a significant impact on *Lactobacillus* spp. (*p* < 0.001) prevalence, indeed the goji group had higher means on day 0 (*p* < 0.05) and on day 4 (*p* < 0.001) than the control group. Lastly, with regards to the consumer’s test, the tasters assigned a higher score to GBs rabbit meatballs and the purchase interest increased when the rabbit diet was known. Overall, these results indicate that the goji berries inclusion in the rabbit diet could represent a valuable strategy to improve quality and sensory traits of meat.

## 1. Introduction

Goji berries (GBs) (*Lycium barbarum*) are grown naturally in East Asia, especially in the valleys of the Himalayas [1]. GBs are historically considered an essential element in the Mongolian and Tibetan traditional medicine, generically used as a dietary supplement [2]. *L. barbarum* was initially introduced in Europe in the 16th century as an ornamental tree, and only after the 21st century was its fruit (GBs) included into Western diets [3].

Some authors [4,5] reported the valuable nutritional characteristics of goji berries, since they contain various vitamins (particularly riboflavin, thiamine, and ascorbic acid), minerals, amino acids, and a complete spectrum of antioxidant carotenoids. Its health promoting properties were classified as nutraceutical food [5], used in human dietary as prevention of diseases such as diabetes, cardiovascular diseases, and for the reduction of cancer risk [6]. The goji berries health benefits are also represented by their protection of the gastrointestinal tract as well as their anti-ageing and immune-stimulating properties [1].

These properties make them a good antioxidants supplement in diets or humans as well as for animals [7]. Many studies have, in fact, been conducted in order to investigate the biological activities of goji berries, although mostly laboratory animals (mice and rats) have been used [8].

Several authors [9,10] have highlighted the beneficial effects of GBs supplementation on the animal gut microbiota due to the presence of functional components, including polysaccharides, which play an important role in promoting the growth of probiotics, such as bifidobacteria and lactobacilli. Wang et al. [11] showed that a diet with a 1% *w*/*w* of goji berries supplement administered for 10 weeks to female IL-10-deficient mice, led to a decrease in the contents of ω-6 Polyunsaturated Fatty Acids (PUFAs), an inhibition of urease activity and to anabolism of amino acids in the colon contributing to the animal gut epithelial health.

Another important aspect examined by different authors [12,13,14,15,16] are the benefits of adding different categories of bioactive molecules, mainly extracted from plants, including goji berries, to the food, to improve the microbiological, chemical, and sensory quality. Specifically, Rotar et al. [17] showed that the addition of a goji berries supplement (7% *w*/*w*) to classic yoghurt resulted in an increase in lactic acid bacteria (LAB), maintaining the prebiotic value of yoghurt during storage. Moreover, yoghurt with 7% GBs supplement, obtained better results in the consumer test.

Some authors [18,19,20], suggested that the beneficial and nutritional properties of goji berries are able to express their action not only at the level of the human or laboratory animals’ microbiota, but also on livestock animals. Menchetti et al. have previously conducted studies on does and growing rabbits evaluating the effect of the GBs supplementation on the productive performance [21] and meat quality [22]. Specifically, they showed that goji berries inclusion in the feed reduced pre-weaning mortality, improved the feed conversion rate on growing rabbits, and increased milk yield of the does. The same authors have also recently shown that GBs supplementation increased phenolic content and antioxidant properties of rabbit meat. However, they did not analyze the effect of supplementation on body weight (BW) of adult animals nor the changes in the microbiological profile of the meat during storage.

The possibility to improve the microbiological and organoleptic meat quality from a point of view of animals at the end of their reproductive life through the use of new biological compounds, represents a sustainable strategy that could allow to relaunch the rabbit meat sector [23]. Even though the healthy characteristics of rabbit meat are undisputed, the industry is going through a period of crisis due to reductions in consumption and welfare issues [24]. The use of nutraceutical products in breeding could be a marketing strategy that also meets the needs of animal welfare [21]. Furthermore, the rabbit is an excellent animal model for other species [25,26] that could benefit from the positive effects of goji berries.

Therefore, given that the effects of GBs supplementation on microbiological quality and on sensory characteristics of foods remained unexplored to this point, the aim of the study was to analyze the impact of dietary supplementation with 3% (*w*/*w*) of goji berries on microbiological quality, physicochemical, and sensory characteristics of rabbit meat.

## 2. Materials and Methods

### 2.1. Animals and Feeding

The trial was carried out at the Department of Agricultural, Food, and Environmental Science of the University of Perugia’s experimental farm. Rabbits were maintained in a controlled condition: temperature ranged from 17 to 22 °C, relative humidity of 60% and a continuous photoperiod of 16 h light per day. Fresh water was always available. The study was conducted in accordance with the Legislative Decree No. 146, implementing Directive 98/58/EC of 20 July 1998 concerning the protection of animals kept for farming purposes.

Forty-two New Zealand White multiparous (one year old) were randomly assigned to two groups (*n* = 21/group) on the basis of the dietary treatment: the control group was fed using a commercial pelleted feed, and the goji group was fed the same feed but supplemented with 3% goji berries (Table 1). Goji berries were provided by Impresa Agricola of Gianluca Bazzica, Foligno (Italy) in dried form. They were then ground into smaller pieces, but not into powder, and mixed together with the rest of the diet’s ingredients Finally, the feed was pelleted through a pellet machine present in the farm and after drying, it was ready for use. During the experimental period (105 days, from March to July 2020) the animals received 130 g/d of feed and were weighed weekly during the dietary treatment as well as before and after slaughtering. At slaughter, the carcass yield was calculated as ((dressed carcass weight/live weight) × 100).

### 2.2. Rabbit Meat Microbiological Parameters

Immediately after slaughtering, 84 loins (Longissimus thoracis et lumborum), belonging to 42 rabbits, were isolated and were individually randomly packed in an oxygen-permeable package consisting of expanded polystyrene tray covered with PVC film. After packing, all the samples were transported to the laboratory of Food Inspection at Department of Veterinary Medicine, University of Perugia (Italy), under refrigerated conditions and subsequently the microbiological analyses were performed on the rabbit loins samples (14 per group) at 6 h post mortem (day 0), after 4 and 10 days of refrigerated storage (4 ± 1 °C) in dark condition.

The hygiene and food safety parameters on rabbit meat were investigated during storage, aseptically collecting 10 g from each loin and mixing it with 90 mL of sterile buffered peptone water (Oxoid, Basingstoke, UK). After homogenization in a Stomacher 400 (Stomacher 400 circulator; Seward Ltd., Norfolk, UK) for 60 s, decimal dilutions were prepared for the subsequent determinations.

*Enterobacteriaceae*, *Escherichia coli*, total coliforms and coagulase positive staphylococci were enumerated using Petrifilm (3M, St. Paul, MN, USA), following the AFNOR 3M 01/06-09/97, AFNOR 3M 01/08-06/01, AFNOR 3M 01/2-09/89 A and AFNOR 3M 01/09-04/03 A methods, respectively. The count of *Pseudomonas* spp. was performed using *Pseudomonas* Agar Base (Biolife Italiana s.r.l., Milan, Italy) with CFC *Pseudomonas* Supplement (Biolife), incubated at 25 °C for 48 h. For the mesophilic aerobic bacteria, Plate Count Agar (Oxoid, Basingstoke, UK) was aerobically incubated at 30 °C for 48 h. *Lactobacillus* spp. were counted on de Man, Rogosa and Sharpe agar (Biolife) anaerobically incubated at 37 °C for 48 h, while *Lactococcus* spp. on M17 Agar (Biolife) aerobically incubated at 37 °C for 48 h. Moreover, *Salmonella* spp. detection and *Listeria monocytogenes* count (analytical unit: 25 g) were carried out according to UNI EN ISO 6579-1:2017 and AFNOR BRD 07/05-09/01, respectively.

The analyses were performed in duplicate and the results were expressed as Log CFU/g.

### 2.3. Rabbit Meat Physico-Chemical Measurements

At each time considered, on 14 loins per group the pH was measured in triplicate using a pH meter equipped with an insertion electrode (Crison pH25, Crison, Barcelona, Spain). With regards to the color analysis, three measurements were performed on the surface of the loins after transversal cutting of the caudal portion and oxygenation for 5 min at room temperature. Color coordinates, expressed as lightness (L*), redness (a*), and yellowness (b*), were assessed by a colorimeter (Minolta CR400 Chromameter, Osaka, Japan—light source of D65 calibrated against a standard white tile) using the CIE L* a* b* system (CIE, 1976). Differences in lightness (ΔL), redness (Δa), and yellowness (Δb) between groups were calculated for each time. Moreover, the total color difference (ΔE) was calculated as proposed by Sharma [28]:

(1) $$\Delta E_{0-1}=\sqrt{{\left(L_{0}-L_{1}\right)}^{2}+{\left(a_{0}-a_{1}\right)}^{2}+{\left(b_{0}-b_{1}\right)}^{2}}$$

A score of 2.3 was used as threshold for human noticeable difference [28,29].

Lipid oxidation, expressed as mg malondialdehyde (MDA)/kg muscle, was determined using TBARS (thiobarbituric reactive substances) test according to Tarladgis et al. [30]. For the quantification of the nitrogenous compounds in meat, total volatile basic Nitrogen (TVBN) was calculated using VELP Marka model UDK 139 apparatus (Velp Scientifica, Usmate, Milan, Italy). Briefly, the samples (10 g) were alkalized with 2 g of magnesium oxide and the TVBN values were determined by steam distillation and titration with 0.01 N HCl. Only at day 0 meat samples were evaluated for moisture, fat, protein, and ash according to AOAC (Association of Analytical Chemists, 2000) using methods 950.46, 960.30, 992.15, and 923.03, respectively.

### 2.4. Rabbit Meat Sensory Analysis

A consumer panel was performed at Goodmen.it S.r.l., Perugia (Italy) and was carried out in a space set up in the open air respecting the safety measures on social distancing imposed by the COVID-19 emergency. A group of regular meat consumers (60 tasters) have completed a questionnaire which included the following information: age, sex, and the frequency with which they consume rabbit meat. The assessors provided their consent before test sections, stating they did not receive any incentives for their participation and the questionnaires were returned anonymously. No ethical approval was requested. For the preparation of samples, rabbit posterior thighs, after 1 month of frozen storage (−18 °C), were thawed for 24 h at 4 °C and deboned. The meat then was minced and used for preparing meatballs (45 g each). The meatballs were cooked in the oven (Electrolux, air-o-steam^®^ Touchline), without any salt and spices, at 140 °C (10% relative humidity) for 30 min and they were kept warm until served. Before the consumer test was carried out, a training session was performed to explain the use of the hedonic scale made of nine points, which ranged from 1 = dislike extremely to 9 = like extremely. Single meatballs were served on white plastic plates identified by an ID number specific for control and goji group. Assessors were asked to rate sensory attributes using a nine-point hedonic scale for color, taste, juiciness, and overall liking. Purchase intent was evaluated using the binomial (yes/no) scale before and after acquiring information about dietary supplementation of the rabbit.

### 2.5. Data Analysis

Statistical distribution of microorganisms in rabbit meat was expressed as both prevalence (percentage of units that contain the target organism above a predetermined microbiological limit) and microbial count (expressed as Log CFU/g) [31].

First, the proportion of samples in which mesophilic aerobic bacteria, *Lactococcus* spp., *Lactobacillus* spp., Enterobacteriaceae, total coliforms, and *Pseudomonas* spp. were not detectable (<1 Log CFU/g) and detectable (>1 Log CFU/g) was analyzed by generalized linear model (GLM) using binomial as probability distributions and logit as link function. Effects of the group (2 levels: control and goji), sampling time (3 levels: 0, 4, and 10 d), and their interaction were evaluated. 

Full factorial models using GLM procedures were also used to evaluate the effects of the group and sampling time on pH, color related parameters, and microbial count. For these variables, normal and identity were respectively set as probability distribution and link function. Only the samples with positive results (>1 Log CFU/g) for each microorganism were considered for this analysis.

In order to evaluate the differences in BW and feed intake, the time effect had 14 levels. With regards to the dressed carcass, weight, and carcass yield, only the effect of group was included in the GLMs. Diagnostic graphics were used to check assumptions and outliers. Pairwise comparisons were performed using the least significant difference. The *p* values from Wald chi-square tests were reported. Results were expressed as estimated marginal means ± standard error (SE) while row data were presented in figures. Data obtained from the consumer test were analyzed using GLM procedure of SAS testing the group (control and goji) as fixed factor.

Consumer’s willingness to purchase was compared (i) between the two groups by chi-square test, and (ii) between before and after information (within each group) by McNemar test.

Statistical analyses were performed with SPSS Statistics version 25 (IBM, SPSS Inc., Chicago, IL, USA). Statistical significance occurred when *p* < 0.05.

## 3. Results and Discussion

### 3.1. Productive Performance

All of the rabbits’ food intake decreased in the last few weeks (from 910 ± 14 g/week during the first week to 836 ± 14 g/week during the last week; *p* < 0.001), probably due to the effects of the warm season. However, no differences between groups (885 ± 5 g/week and 898 ± 6 g/week for control and goji, respectively; *p* = 0.099) were recorded. Rabbits’ mean BW during the dietary treatment was higher in the goji group (4354 ± 23 g) than the control group (4278 ± 22 g; *p* = 0.016), although the weights at slaughtering did not differ (4310 ± 82 g and 4357 ± 82 g for control and goji group, respectively; *p* = 0.634). There were no differences between groups in either dressed carcass weights (2463 ± 56 g and 2471 ± 58 g for control and goji, respectively; *p* = 0.922) or carcass yield (57.2 ± 0.6% and 56.6 ± 0.9% for control and goji, respectively; *p* = 0.442). These findings appear to differ from what is reported by Menchetti et al. [21] which found increased growth rates in rabbits supplemented with goji berries. This result could be due to the different physiological moment of the animals used in our study (end of career) compared to those used in the previous one (rabbits in growth). However, diets supplemented with natural bioactive compounds have not always had an effect on animal growth or on the improvement of the yield at slaughter, as reported by Bai et al. and Koné et al. [20,32].

### 3.2. Color Values and pH 

The mean pH was 5.9 ± 0.1 for both groups at all times (*p* = 0.710). Therefore, no significant effect was found, in accordance with what was previously reported by Menchetti et al. and Cullere et al. [22,24].

Overall, in our study the a* values increased at the end of storage (from 0.18 ± 0.19 at day 0 to 1.78 ± 0.19 at day 10; *p* < 0.001) while a reduction in L* value was found after four days of storage (from 51.2 ± 0.5 at day 4 to 49.6 ± 0.5 at day 10 of storage; *p* = 0.028). These results in part overlap with what was reported by Smeti et al. and Nieto et al. [33,34], who observed a decrease in L* values and an increase with a subsequent decrease in a* values during refrigerated storage of lamb and ewe meat. Conversely, Cullere et al. [24] found no variation during shelf life in L*, a*, and b* coordinates on rabbit meat, regardless of the type of diet and packaging. In the current work, b* values showed a fluctuating trend in both groups as it decreased after four days (from 0.76 ± 0.14 at day 0 to −0.02 ± 0.14 at day 4; *p* < 0.001) and increased in the last observation (1.25 ± 0.14; *p* = 0.014). It is important to specify that the color variations in the meat depend on many variables, not only on the type of animal and the diet, but also on the anatomical cut, amount of fat or packaging type [29,35,36]. For these reasons it is not easy to compare the results in the literature with those obtained in this study.

As for the influence of the diet, some changes in color parameters were noteworthy. Firstly, the control was lighter than goji group (51.2 ± 0.4 and 49.9 ± 0.4 for L* marginal means of control and goji groups, respectively; *p* = 0.032) although multiple comparisons indicated significant differences only at day 0 (52.3 ± 0.7 and 49.6 ± 0.7 for control and goji groups, respectively). Secondly, a significant effect of the group × time interaction (*p* = 0.01) was found for b* values. However, pairwise comparisons found greater values in the control (1.1 ± 0.2) than in the goji groups (0.4 ± 0.2; *p* = 0.012) only at day 0. Subsequently, the values followed fluctuating trends in both groups, the differences were not significant, and the values very variable. The more yellowish color in the control group, even if only at day 0, could be due to a greater lipid oxidation that has developed in the meat of rabbits not fed with a diet supplemented with natural antioxidant substances and rich in polyphenols [34,37].

Generally, the color variations in meat and meat preparation during shelf life are manly influenced by oxidative processes of both protein and lipid component [24].

Indeed, several studies [34,38,39] reported that polyphenols have a positive role in stabilizing the meat color due to the delaying effect on lipid oxidation.

**Figure 1:**
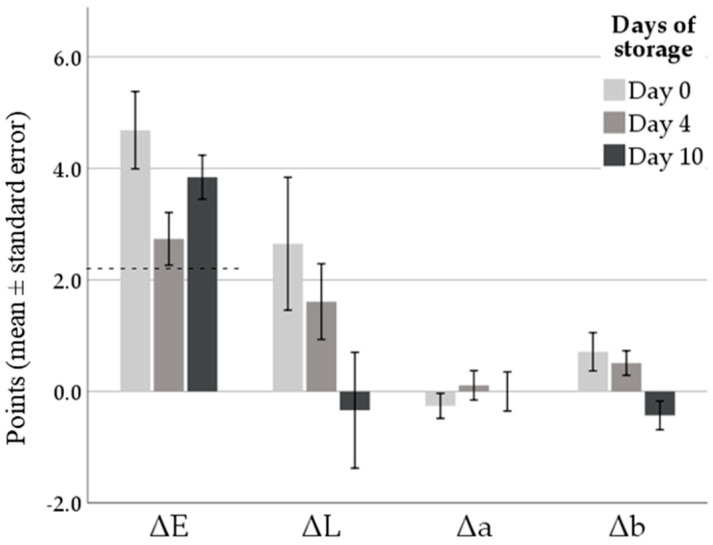
Effects of Goji berries inclusion in the diet of rabbits on meat color. Total color difference (ΔE) and differences in lightness (ΔL), redness (Δa), and yellowness (Δb) between groups (control–Goji) at slaughtering and at day 4 and 10 of shelf-life. The dashed line indicates the threshold of human noticeable differences for (>2.3 points) for ΔE.

Overall, these differences lead to a ∆E (total color difference) higher than 2.3 points (Figure 1) highlighting that the human eye at visual color appraisal perceives differences between the two groups in all three times. Perceptible color differences can have an important impact on consumer in stimulating more agreeable perception of the appearance of one meat compared to another [40].

### 3.3. TBARS and TVBN

Goji supplementation also affected lipid oxidation of rabbit meat. Indeed, overall TBARS values increased progressively during storage (*p* = 0.042), although the goji group showed lower values than control on all observation days (*p* < 0.001; Table 2).

TBARS are formed as a by-product of lipid peroxidation and can be strongly related to the lipid composition of the meat (Table S1). The obtained results, as reported by several authors [2,22], highlight the positive relationship between dietary supplementation with GBs and the decrease in lipid oxidation in rabbit loins. Moreover, this result was in concordance with our above-mentioned results regarding the b* values where the loins belonging to the control group appeared to be more yellowish, indicator of increased lipid oxidation. However, all the samples in all three analysis fall widely within the threshold reported for TBARS of meat (2–2.5 mg/kg) [41].

The TVBN values had the same trend over time (*p* < 0.001). A significant group effect was also found (*p* = 0.024); however, goji group had higher values than the control at day 0 (*p* = 0.010; Table 2). The TVBN values are used as reference index to evaluate the freshness level in perishable food, especially in fish and meat [42]. Generally, TVBN is influenced and increased by the activities of endogenous enzymes and spoilage bacteria [43]. Our results showed a slight difference only at day 0 for the goji group with marginally higher TVBN values than control samples. For the following times, the differences were not significant and the TVBN values for both groups were overlapping. 

Differently from fish, where there are reference values in relation to different categories of fishery products (Reg. (EC) No 2074/2005), there are no TVBN reference limits for meat. However, Byun et al. [44] have proposed, for beef and pork meat, TVBN limit values of about 20 and 30 mg N/100 g, while Pearson et al. [45] defined a TVBN value for hygienic standard for livestock meat stored at refrigeration temperature of ≤20 mg N/100 g. Furthermore in this case, the obtained results in our study are compliant to the published values, for both groups and throughout the shelf life.

### 3.4. Meat Microbial Status 

**Figure 2:**
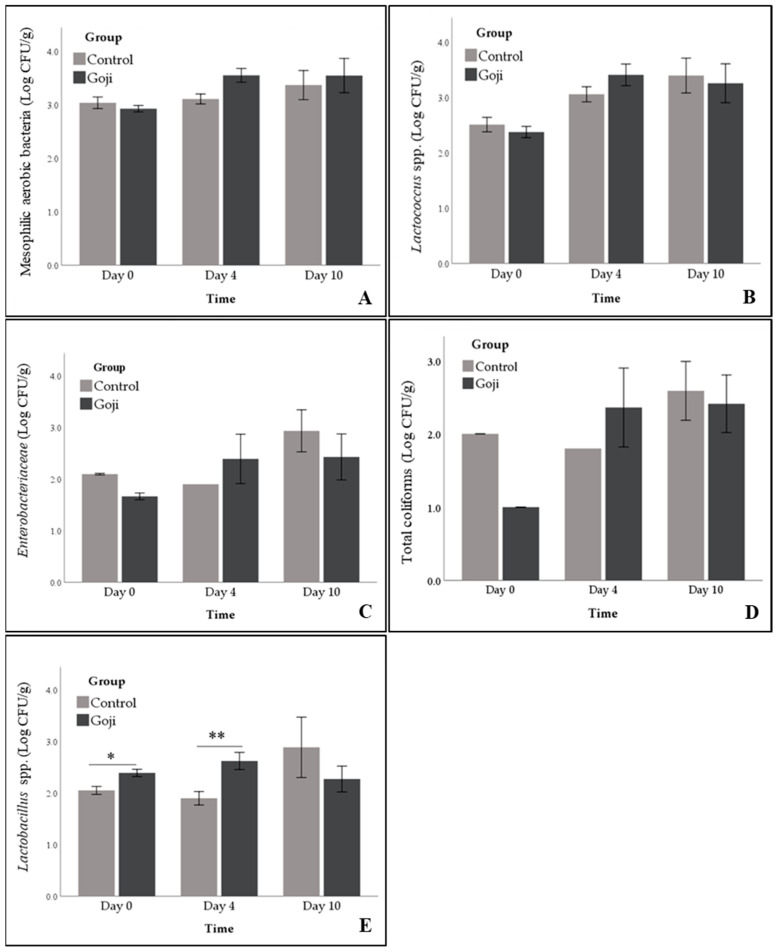
Effects of Goji berries inclusion in the diet of rabbits on counts of mesophilic aerobic bacteria (**A**), *Lactococcus* spp. (**B**), Enterobacteriaceae (**C**), total coliforms (**D**), and *Lactobacillus* spp. (**E**) on meat at slaughtering (Day 0), day 4, and 10 of shelf-life. The bar graphs show mean ± standard error. Anomalies in the error bars are due to the low prevalence. *: *p* < 0.05; **: *p* < 0.001.

Colonies (>1 Log CFU/g) of mesophilic aerobic bacteria were observed in all samples (100.0 ± 0.0%) and their count increased over time, regardless of their group (from 2.9 ± 0.1 at day 0 to 3.4 ± 0.1 Log CFU/g at day 10; *p* = 0.026; Figure 2A).

**Figure 3:**
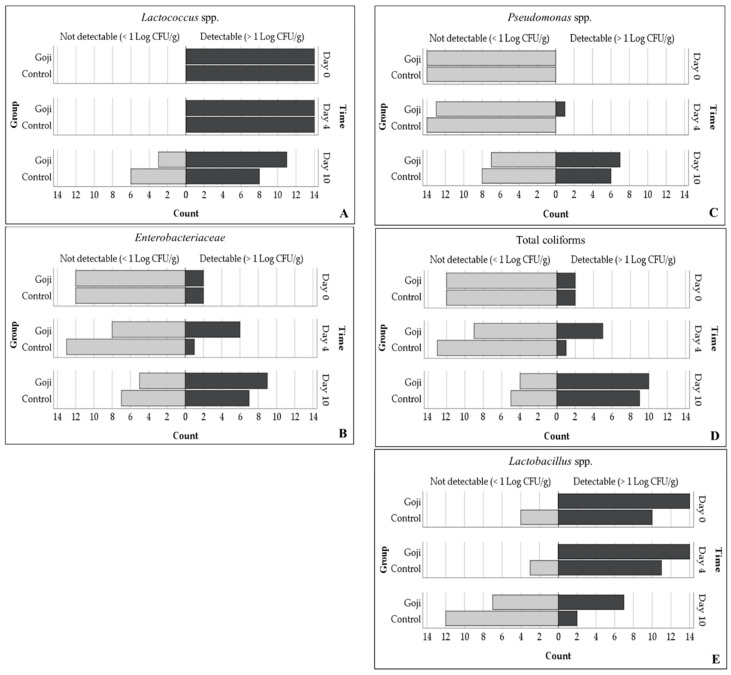
Population pyramids that show the absolute number of rabbit’s meat units containing colonies of *Lactococcus* spp. (**A**), Enterobacteriaceae (**B**), *Pseudomonas* spp. (**C**), total coliforms. (**D**), and *Lactobacillus* spp. (**E**). The limit of detection was 1 Log CFU/g.

The number of positive plates for *Lactococcus* spp. reduced from 100.0 ± 0.0% at day 0 to 68.9 ± 9.1% at day 10 (*p* = 0.001; Figure 3A). Moreover, overall, their count (in positive samples) increased progressively from 2.4 ± 0.1 Log CFU/g at day 0 to 3.3 ± 0.2 Log CFU/g at day 10 (*p* < 0.001; Figure 2B). However, also for the lactococci, the effect of the dietary treatment was not significant (*p* = 0.868).

The prevalence of *Enterobacteriaceae* increased from 14.3 ± 6.6% at day 0 to 57.3 ± 9.5% at day 10 (*p* < 0.01; Figure 3B) but no difference between groups were found (37.8 ± 8.5% and 19.0 ± 7.1% for goji and control group, respectively; *p* = 0.105; Figure 3B). Instead, their numerical counts were influenced neither by the group nor by time (Figure 2C).

After slaughtering, *Pseudomonas* spp. was not detected (0.0 ± 0.0%) but the prevalence increased until 46.4 ± 9.4% at day 10 (*p* < 0.001). At day 4 it was detectable only in one sample (2.7 Log CFU/g) while at day 10 in 13 samples with concentration of 2.7 ± 0.41 Log CFU/g and 2.96 ± 0.54 Log CFU/g in goji and control groups, respectively (Figure 3C). Therefore, no difference between groups was found, neither in numerical counts, nor in prevalence (*p* = 0.704).

Similarly, no differences were detected for total coliforms counts (*p* = 0.702; Figure 2D) but only a significant increase in prevalence after 10 days of storage was found (from 14.3 ± 6.6% at day 0 to 68.0 ± 8.9% at day 10; *p* < 0.001; Figure 3D).

All samples were negative for *Salmonella* spp., *L. monocytogenes* and coagulase-positive staphylococci.

The obtained results from the microbiological analyses described above, have shown that there are no differences between the two groups and these results lead us to state that diet supplementation with compounds rich in natural antioxidants does not improve the meat’s hygienic health profile, as reported by other authors [24,46]. Moreover, the small number of samples in which Enterobacteriaceae, *Pseudomonas* spp., and total coliforms were detectable, and their considerable variability, could contribute to the lack of significant effects.

Generally, bacterial contaminations once present, can grow or remain stable during meat shelf life [47]. These contaminations can occur in stages during and after slaughter and can be caused by bacteria normally present in animal gut, slaughterhouse environment or by dirty equipment that can contaminate the carcasses, the subsequent cuts and processed meat products [47,48]. Considering that microbial safety has assumed significant importance among the factors that determine meat quality, the use of integrated diets with natural products can represent a simple and sustainable strategy to improve meat quality. Indeed, other authors have evaluated the effects of different diets supplemented with natural compounds on the microbiological profile of rabbit meat. Vannini et al. [49] showed that a supplementation with whole linseeds limited bacterial growth in rabbit meat, extending the shelf life. The same author reported a similar action of bacterial inhibition by an integrated diet with high percentages of dehydrated alfalfa meal [50]. However, our study showed that the supplementation with GBs enhanced the growth of the meat lactic acid bacteria, even though detectable *Lactobacillus* spp. reduced from 100.0 ± 8.5% at day 0 to 29.0 ± 9.6% at day 10 (*p* < 0.001; Figure 3E). Indeed, significant group effects were found since the estimated marginal means of the samples with detectable *Lactobacillus* spp. were higher in the goji than in the control group (100.0 ± 0.3% and 53.5 ± 9.7% for goji and control group, respectively; *p* < 0.001). In particular, pairwise comparisons showed higher prevalence for goji group at day 0 (100.0 ± 0.0% and 71.4 ± 12.1% for goji and control group, respectively; *p* = 0.018) and day 4 (100.0 ± 0.0% and 78.6 ± 11.0% for goji and control group, respectively; *p* = 0.050; Figure 3E). Moreover, significant effect of group x time interaction was found for their count (*p* = 0.002): Goji had higher means at day 0 (*p* = 0.041) and day 4 (*p* < 0.001) than the control group. These differences were not significant at day 10, when a low number of positive samples and a greater variability of the data can be noted (Figure 2E).

Several authors [51,52,53] have reported the beneficial effects of supplementing the diet with goji berries on the animal gut by acting on the intestinal microbiota. In our study, the influence on microbiota could have had an impact on the lactobacilli of the rabbit meat. In fact, the GBs dietary supplement was able to increase the prevalence and the number of these bacteria in meat samples from animals fed with goji berries.

The role of lactic acid bacteria in the fresh meat is controversial, because certain species can generate offensive metabolites that increase the organoleptic deterioration of meat, while other LAB strains act as bioprotective agents and play an important role during storage, carrying out a competitive action against spoiling and pathogenic bacteria [54,55]. In our case, it was not possible to evaluate the actual inhibitory activity carried out by lactobacilli because *Salmonella* spp. and *L. monocytogenes* were absent in both groups and at all times of analysis. The same results were obtained for spoilage microorganisms and hygiene indicator bacteria. Indeed, until the end of storage (day 10), these bacteria have remained at satisfactory levels as reported by CeIRSA (Centro Interdipartimentale di Ricerca e Documentazione sulla Sicurezza Alimentare) [56] for the food category “fresh meat and meat product” (sub-point 9.1: “fresh refrigerated meat”). These results lead us to conclude that further studies are needed to establish whether supplementation with GBs can increase LAB to such an extent as to contrast the development of unwanted bacteria.

### 3.5. Sensory Evaluation

As shown in Table 3, differences among control and goji meatballs were found for juiciness, taste and overall liking, while no difference were registered for color. In particular, goji meatballs were perceived by the tasters juicier than control and this evaluation could also have influenced the taste and overall liking. This increased juiciness in goji samples could be linked with the presence of antioxidants in meat which are able to enhance water holding capacity [57,58]. Furthermore, the consumers showed increased interest in the purchase of goji meatballs in the informed session. Indeed, after giving the information about dietary treatment, the purchase intent of goji meatballs showed an increase (*p* < 0.01) while the purchase intent of the control samples did not show significant changes (*p* = 0.180). In our opinion the consumers were positively affected by the information, showing that the choice can be influenced by product information. These results suggest that the inclusion of goji berries in the rabbit diet could be a good marketing strategy perhaps because the consumers appreciate the use of nutraceutical product also in animal nutrition. Moreover, several studies [37,48] on consumers choice demonstrated how the effect of the information can be able to modify consumer behavior.

## 4. Conclusions

The incorporation of 3% *w*/*w* of goji berries in the rabbit diet was able to increase the lactobacilli population, decrease the lipid oxidation and improve the sensory traits of rabbit meat. However, the diet integration, did not implement the hygienic profile of the rabbit meat. In this regard, the data in the literature concerning the intake of bioactive compounds through the diet as well as their antimicrobial activity in the meat and meat products are still rather contrasting. Presumably, the multiple variables involved, such as the type of dietary enrichment, the percentage of supplementation, the animal species used, the hygienic level of the meat and the microorganisms involved, do not allow for univocal results.

Nevertheless, GBs supplementation could represent a feasible strategy to improve some physicochemical aspects and sensory characteristics, while simultaneously increasing the consumer’s predisposition to purchase. Definitely, further studies are needed to define (i) lactobacilli biotypes to understand their role during the rabbit meat shelf-life; and (ii) the mechanism of absorption and integration into the meat structure of the bioactive molecules contained in goji berries and their influence on carcass microbial population, in order to take full advantage of GBs antioxidant and probiotic properties.

## Figures and Tables

**Table 1:** Formulation and chemical composition (as fed) of control and experimental diet supplemented with goji berries.

	Unit	Group	
		Control	Goji
**Ingredients**			
Wheat bran	%	30.0	29.0
Dehydrated alfalfa meal	%	42.0	41.0
Barley	%	9.5	9.0
Sunflower meal	%	4.5	4.2
Rice bran	%	4.0	3.9
Soybean meal	%	4.0	3.9
Calcium carbonate	%	2.2	2.2
Cane molasses	%	2.0	2.0
Vitamin–mineral premix ^1^	%	0.4	0.4
Soybean oil	%	0.4	0.4
Salt	%	0.3	0.3
Goji berries	%	-	3.0
**Analytical data**			
Crude Protein	%	15.74	15.66
Ether extract	%	2.25	2.47
Ash	%	9.28	9.25
Starch	%	16.86	16.99
NDF ^3^	%	38.05	37.49
ADF ^3^	%	19.54	19.01
ADL ^3^	%	4.01	3.98
Digestible Energy ^2^	MJ/kg	10.3	10.3

^1^ Per kg diet: vitamin A 11,000 IU; vitamin D3 2000 IU; vitamin B1 2.5 mg; vitamin B2 4 mg; vitamin B6 1.25 mg; vitamin B12 0.01 mg; alpha-tocopherol acetate 50 mg; biotine 0.06 mg; vitamin K 2.5 mg; niacin 15 mg; folic acid 0.30 mg; D-pantothenic acid 10 mg; choline 600 mg; Mn 60 mg; Fe 50 mg; Zn 15 mg; I 0.5 mg; Co 0.5 mg. ^2^ Estimated by Maertens et al. [27]. ^3^ NDF: neutral detergent fiber; ADF: acid detergent fiber; ADL: acid detergent lignin.

**Table 2:** Effects of Goji berries inclusion in the diet of rabbits on thiobarbituric reactive substances (TBARS) and Total Volatile Basic Nitrogen (TVBN) at different days of storage (mean ± standard error).

Parameter	Time (Day of Storage)	Group		*p* Value		
		Control	Goji	Group	Time	Group × Time
**TBARS** **(mg MDA/kg)**	Day 0	0.248 ^aA^ ± 0.048	0.135 ^bA^ ± 0.027	<0.001	0.042	0.257
	Day 4	0.398 ^aB^ ± 0.050	0.144 ^bA^ ± 0.028			
	Day 10	0.374 ^aB^ ± 0.072	0.232 ^bA^ ± 0.045			
**TVBN** **(mg N/100 g)**	Day 0	14.919 ^aA^ ± 0.327	15.823 ^bAB^ ± 0.336	0.024	<0.001	0.204
	Day 4	15.140 ^aA^ ± 0.160	15.584 ^aA^ ± 0.151			
	Day 10	16.300 ^aB^ ± 0.339	16.320 ^aB^ ± 0.329			

^a,b^ Values followed by the same letter in each row do not differ significantly (*p* < 0.05; multiple comparisons using the least significant difference). ^A,B^ Values followed by the same letter in each column do not differ significantly (*p* < 0.05; multiple comparisons using the least significant difference).

**Table 3:** Mean consumer scores for sensory acceptability and purchase intent of rabbit meat balls.

Attributes	Group		SEM	*p* Value
	Control	Goji		
Color	5.81	5.93	0.152	0.590
Juiciness	5.58	6.42	0.141	<0.01
Taste	6.17	6.68	0.153	0.020
Overall liking	6.08	6.50	0.150	0.042
**Positive (Yes) Purchase Intent (%)**				
Before information	31.7	45.0	-	0.133 *
After information	21.7	71.7	-	<0.001 *

Mean of 60 consumers based on a 9-points hedonic scale (1 = dislike extremely, 9 = like extremely); SEM, standard error of least square means; purchase intent was obtained from both before and after consumers had gained information about the rabbit diet. * from chi-square test.

## Supplementary Materials

The following are available online at https://www.mdpi.com/2304-8158/9/10/1480/s1, Table S1. Centesimal composition of “Longissimus thoracis et lumborum” muscle in control group and supplemented with 3% of Goji (mean ± standard deviation).
