# Supplementary material for: Impact of Dietary Supplementation with Goji Berries (Lycium barbarum) on Microbiological Quality, Physico-Chemical, and Sensory Characteristics of Rabbit Meat
Source: Foods. 2020 Oct 16;9(10):1480. doi: 10.3390/foods9101480 (PMC7603015; doi:10.3390/foods9101480)
Supplement: Supplementary file 1 [file foods-09-01480-s001.pdf]

Table S1. Centesimal composition of *Longissimus thoracis et lumborum* muscle in control group and supplemented with 3% of Goji (mean ± standard deviation).

	**Group**	
	**Control**	**Goji**
Moisture (%)	74,68 ±1,37	74,54 ±1,11
Crude protein (%)	21,55 ±0,38	21,60 ±0,35
Ether extract (%)	2,59 ±0,10	2,73 ±0,21
Crude ash (%)	1,18 ±0,02	1,13 ±0,03
